# Disparities in Hypertension Prevalence, Awareness, Treatment and Control between Bouyei and Han: Results from a Bi-Ethnic Health Survey in Developing Regions from South China

**DOI:** 10.3390/ijerph13020233

**Published:** 2016-02-19

**Authors:** Fen Dong, Dingming Wang, Li Pan, Yangwen Yu, Ke Wang, Ling Li, Li Wang, Tao Liu, Xianjia Zeng, Liangxian Sun, Guangjin Zhu, Kui Feng, Biao Zhang, Ke Xu, Xinglong Pang, Ting Chen, Hui Pan, Jin Ma, Yong Zhong, Bo Ping, Guangliang Shan

**Affiliations:** 1 Department of Epidemiology and Biostatistics, Institute of Basic Medical Sciences Chinese Academy of Medical Sciences, School of Basic Medicine Peking Union Medical College, Beijing 100005, China; fionarab@163.com (F.D.); panli1716@163.com (L.P.); wangkehope@126.com (K.W.); wangli0528@vip.sina.com (L.W.); zxj28@sohu.com (X.Z.); zhuguangjinpumc@126.com (G.Z.); fengkui@sina.com (K.F.); sljzhangbiao11@126.com (B.Z.); 2 Guizhou Center for Disease Control and Prevention, Guizhou 550004, China; wangdingm123@sina.com (D.W.); yuyangweny@163.com (Y.Y.); gpicdp@163.com (L.L.); liutao9099@163.com (T.L.); slx1087@163.com (L.S.); 3 Department of Endocrinology, Peking Union Medical College Hospital, Chinese Academy of Medical Sciences & Peking Union Medical College, Beijing 100730, China; zjwzxuke@126.com (K.X.); pxl19870610@163.com (X.P.); panhui20111111@163.com (H.P.); 4 Department of Ophthalmology, Peking Union Medical College Hospital, Chinese Academy of Medical Sciences & Peking Union Medical College, Beijing 100730, China; ct19870629@hotmail.com (T.C.); majin1912@163.com (J.M.); yzhong_eye@163.com (Y.Z.); 5 Longli Center for Disease Control and Prevention, Guizhou 551200, China; gzlljkpb@126.com

**Keywords:** hypertension, ethnicity, prevention, control

## Abstract

Hypertension is highly prevalent in low-income population. This study aims to investigate ethnic disparities in hypertension and identify modifiable factors related to its occurrence and control in developing regions in South China. Blood pressure was measured in the Bouyei and Han populations during a community-based health survey in Guizhou, 2012. A multistage stratified sampling method was adopted to recruit Bouyei and Han aged from 20 to 80 years. Taking mixed effects into consideration, multilevel logistic models with random intercept were used for data analysis. The prevalence rates of hypertension were 35.3% for the Bouyei and 33.7% for the Han. Among the hypertensive participants, 30.1% of the Bouyei and 40.2% of the Han were aware of their hypertensive conditions, 19.7% of the Bouyei and 31.1% of the Han were receiving treatment, and only 3.6% of the Bouyei and 9.9% of the Han had their blood pressure under control. Age-sex standardized rates of awareness, treatment, and control were consistently lower in the Bouyei than the Han. Such ethnic disparities were more evident in the elderly population. Avoidance of excessive alcohol consumption and better education were favorable lifestyle for reduction in risk of hypertension. Moderate physical activity improved control of hypertension in Bouyei patients under treatment. Conclusively, hypertension awareness, treatment, and control were substantially lower in Bouyei than Han, particularly in the elderly population. Such ethnic disparities indicate that elderly Bouyei population should be targeted for tailored interventions in the future.

## 1. Introduction

High blood pressure (HBP) is a major risk factor for coronary heart disease [1] and can initiate a chain of events leading to stroke and coronary heart disease [2]. HBP causes 51% of stroke deaths and 45% of heart disease deaths worldwide [3], which have also been the leading causes of deaths in China [4]. However, hypertension is preventable and controllable. Since most hypertensive people experience no symptoms at all, everybody should know their BP readings [3].

An increasing number of studies on hypertension have been carried out in the medium- or low-income countries [5,6], showing that highly prevalent hypertension is posing a big health burden on the low-income populations. China, the most densely populated country with multi-ethnic sub-populations, experiences its economic growth with great geographic differences [7] between its relatively wealthy east coast and the under-developed southwest rural interior areas. Guizhou, located in the southwest of mainland, is an economically developing province with residents mainly (66.19%) living in rural settings [8]. It has diverse ethnic groups that offer ideal opportunities for studying the ethnic disparities in health-related issues. Bouyei, (pronounced as Bu Yi), is one of the native minority groups. They are aggregated in two autonomous prefectures and some adjacent cities in the province [8], accounting for almost all of the 2.8 million Bouyei people in China [9]. Studies on hypertension in such a big ethnic group, however, are scarce, let alone on large scales. According to some literatures [10,11,12], prevalence and management of hypertension are different across ethnic groups in China and oversea, but disparities in hypertension between Bouyei and other ethnic groups have not been thoroughly studied. To address this gap, data on blood pressure (BP) in a Health Survey between Bouyei and Han, gathered from a China National Health Survey, was analyzed and hopefully the results could provide evidence for the development of population-based policy as well as effective hypertension prevention and management programs.

## 2. Materials and Methods

### 2.1. Participants

A multistage, stratified, and clustered sampling method was used to select a sample of Bouyei and Han in Guizhou from 27 October to 23 December in 2012. In the first-stage, prefecture-level cities and autonomous prefectures under ethnic minority administration constituted the sampling frame. Based on the economic diversity and high population density of both Bouyei and Han, three areas: Qiannan prefecture, Anshun city, and Guiyang city (capital of the province) were selected, representing the developing, underdeveloped, and most developed cities/prefectures. About 40.2%, 13.8%, and 8.1% of the Bouyei were dispersed in the aforementioned three areas, respectively [8]. In the second-stage, Longli County in Qiannan, Zhenning County in Anshun, and two districts in Guiyang were selected randomly. Within these counties and districts, urban communities or rural townships were selected in the third stage. Of note, the county seat in each county, where the government offices were located, was chosen purposively with the aim to represent the economically developed areas. In the fourth stage, street districts were selected from communities and villages were selected at random from townships. Street districts or villages that had stabilized population were eligible, excluding places with dynamic population or armed forces. Bouyei and Han people aged between 20 and 80 years from the selected places who had stayed there for one year or longer were eligible. They were recruited according to the age-sex-ethnic distribution of Guizhou 2010 Census with their ID cards required for certifying their status. All participants were asked to complete questionnaires through a face-to-face interview and went through a standardized set of measurements. Data from those whose parents were both of Bouyei or Han were analyzed. Participants were excluded for secondary hypertension, cigarette smoking, foods, or alcohol intake before BP was measured, missing information on BP, *etc*. (see online Figure S1 for details in data management).

### 2.2. Measurements

Trained medical staff measured brachial BP when the participant was in the upright sitting position in chairs after a five-minute rest, with the right arm being supported at heart level. Validated automated BP monitors (Omron HEM-907) [13] with suitable cuff were used. Three sequential measurements on BP were taken with one minute apart and the average data was recorded. Information on history of hypertension including prior BP levels and medication use was recorded at the interview.

A 10-mL blood sample was drawn from the antecubital vein in the morning after fasting for over eight hours in a recent week of normal activities and diet. Coagulated blood sample was centrifuged, and serum was segregated and stored in −80 °C freezer for biochemical testing. Waist circumferences were measured with flexible tape at the smallest horizontal circumference between the costal margin and the iliac crest.

Socio-demographic information, personal, and family history of non-communicable diseases and lifestyles were obtained via self-reports. Education was defined as the highest qualification acquired by full-time study and grouped into low (primary school or lower), medium (junior or senior middle school), or high (university or above). Smoking status was classified as never-smokers and ever-smokers, which included current smokers (having been regularly smoking during the previous six months) and ex-smokers (once smoked but had quitted smoking for six months or longer). Alcohol consumption was graded into never/ex-drinker, light drinker (daily ethanol consumption ≤30 mL for men, ≤15 mL for women), and harmful drinker (daily ethanol intake exceeds the levels mentioned above) [14]. Leisure-time physical activity was considered exercising for ≥20 min after work, with three levels as high (5–7 days/week), moderate (1–4 days/week), and low (<1 day/week). Occupational physical activity was grouped into high (sweating and manual work, like farming in the fields), moderate (movement of arms and legs like driving), and low (sedentary or low physical jobs like retail sales). Since physical activity refers to any bodily movement produced by the skeletal muscles that uses energy [15], occupational and leisure-time physical activity were combined to determine the intensity of physical activity as high (vigorous during occupation or leisure time), low (sedentary in both occupational and leisure time), or moderate. Income level was categorized as low (≤250 Yuan), median low (251–800 Yuan), upper middle (801–2000 Yuan), or high (>2000 Yuan), according to the quartiles of individual monthly income. Health insurance and parental history of hypertension were dichotomized into “Yes” or “No”.

### 2.3. Definitions

Hypertension was defined when participants had an average systolic BP (SBP) ≥140 mmHg or diastolic BP (DBP) ≥90 mmHg, or were taking antihypertensive medications. Awareness was defined as whether the hypertensive participants confirmed their morbid status when asked whether they had BP measured before and had a medical diagnosis of hypertension. The treatment was defined as whether the self-reported patients took medication or not. The average SBP and DBP of <140/90 mmHg were the goal levels for BP control in diabetic persons or general people aged <60 years while 150/90 mmHg were for general persons aged ≥60 years [16]. Awareness, treatment, and control are the three aspects to evaluate the effect of hypertension management in population in this study.

### 2.4. Ethics

The study was approved by the Bioethical Committee of the Institute of Basic Medical Sciences, the Chinese Academy of Medical Sciences, Beijing, China (approval No. 028-2013). Informed written consent was obtained from all participants before both physical examination and interview started.

### 2.5. Statistical Analysis

We estimated the sample size for both Bouyei and Han to measure the prevalence rates within 10% precision. Since some other chronic diseases were investigated as well in this survey like diabetes that was less prevalent than hypertension in China, 10% prevalence rate was used for sample size estimation. Considering a 10% lost to follow-up, 10% ineligible sample and the clustered sampling design, the final sample size was nearly 3000 for each of Bouyei and Han populations. All data was independently coded and entered into Epidata (version 3.1) by trained persons in the field. Two sets of independent data input were checked for consistency. Ambiguous information caused by illegible handwriting, contradictory answers in questionnaires, and out-of-range values were resolved at once.

Categorical and continuous variables were represented as numbers (percentages) and median (Interquartile range, IQR), respectively. Proportions or medians between Bouyei and Han were compared using chi-square test or Wilcoxon rank-sum test. Age and sex direct standardization to Guizhou 2010 Census was conducted to compare the hypertension prevalence and rates of awareness, treatment, and control between Bouyei and Han. Based on the multistage stratified sampling design and geographic concentration of ethnic groups, multilevel logistic models with random intercept [17] were used to compare subgroups and identify factors related to prevalence and effective control of hypertension. Urban communities/rural townships were regarded as random intercept in models, taking the mixed effects of different characteristics of clusters and interclass correlation into account. Statistical significance was established at two-tailed *p* < 0.05. All statistical analyses were performed in SAS 9.4 (SAS Institute Inc., Cary, NC, USA).

## 3. Results

### 3.1. Demographic Characteristics

In total, 6073 participants completed questionnaires and health examinations. Finally, data from 2746 Bouyei and 2824 Han were analyzed. The Bouyei participants under study were predominantly rural farmers while Han were mostly urban residents, with majority as females. Compared to Han, Bouyei were much less educated, more likely to drink too much alcohol, and disproportionately less physically active in leisure time, but 58.3% of them were physically exhausted during labor. The income level in Bouyei was much lower than that in Han. More Bouyei had health insurance than Han. Only 11.5% Bouyei reported family history of hypertension, greatly lower than the 31.1% of Han. Bouyei presented lower rates of central obesity and hyperuricemia. They also had significantly higher average SBP. The amounts of fasting glucose, total cholesterol (TC), triglyceride (TG), and low-density lipoproteins cholesterol (LDL), were all consistently lower in Bouyei, along with higher high-density lipoproteins cholesterol (HDL) (all *p* < 0.05 except for sex, smoking status, and DBP) (Table 1).

### 3.2. Prevalence, Awareness, Treatment, and Control

**Figure 1:**
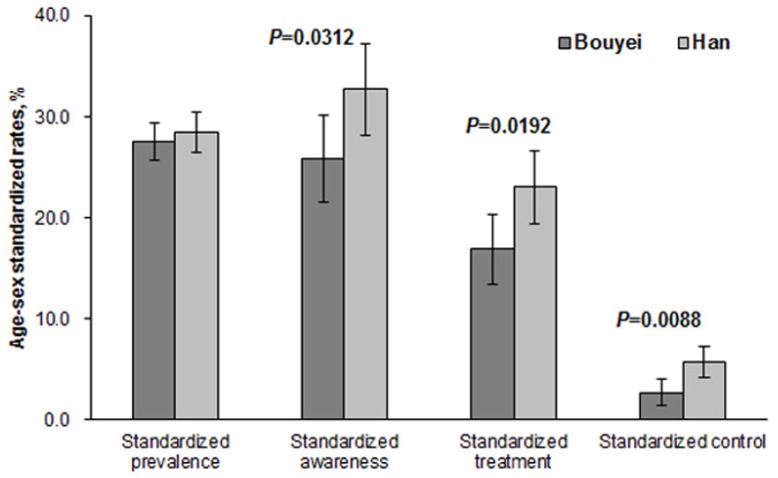
Standardized rates of prevalence, awareness, treatment, and control in Bouyei and Han.

Overall, 35.3% of Bouyei and 33.7% of Han were diagnosed as having hypertension. Among the hypertensive participants, 30.1% Bouyei and 40.2% Han were aware of their hypertensive status, 19.7% Bouyei and 31.1% Han were treated, and 3.6% Bouyei and 9.9% Han had their BP effectively controlled to the recommended targets. The age/sex specific rates of awareness, treatment, and control were consistently lower in Bouyei despite the phenomenon that hypertension was highly prevalent in both ethnic groups (Table 2). The age- and sex-standardized prevalence rates were 27.5% (95% confidence interval (CI), 25.6%–29.4%) in Bouyei and 28.4% (95% CI, 26.5%–30.4%) in Han but the difference was not significant (*p* = 0.5032). The age- and sex-standardized rates of awareness, treatment, and control were significantly lower in Bouyei than those in Han (Figure 1).

**Figure 2:**
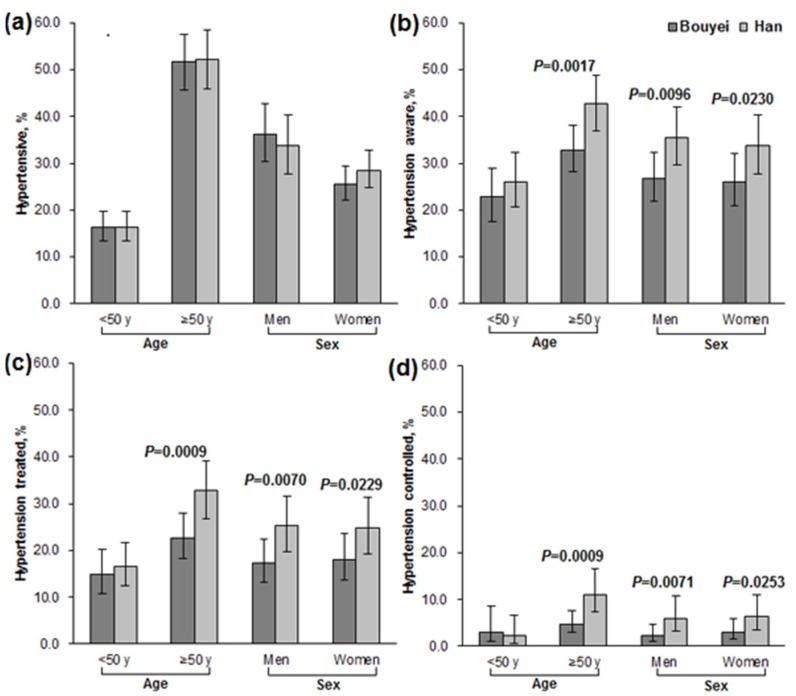
Comparing adjusted prevalence, awareness, treatment, and control by Age and Sex. Error bars indicate limits of 95% CI. (**a**) Adusted prevalence of hypertension in Bouyei and Han; (**b**) Adjusted rates of awareness in hypertensive Bouyei and Han; (**c**) Adjusted rates of treatment in hypertensive Bouyei and Han; (**d**) Adjusted rates of control in hypertensive Bouyei and Han.

Considering the fact that high BP was critically dependent on age and sex, subgroup comparisons were conducted in age/sex strata with clustered effect taken into account (Table S2). Age and sex were mutually adjusted in subgroup comparisons. The adjusted estimates of hypertension prevalence were seen similar between Bouyei and Han in age groups or sex (Figure 2a and Table S2). However, ethnic discrepancies existed in the management of hypertension (Figure 2b,d and Table S2). Bouyei had significantly lower rates of awareness, treatment, and control than Han in both men and women. With respect to age, ethnic discrepancies only existed in the older groups (*p* = 0.0017 for awareness, *p* = 0.0009 for treatment, and *p* = 0.0009 for control), but there were no significant differences in the younger groups, indicating the existence of interactions between ethnicity and age in awareness, treatment, and control of hypertension.

### 3.3. Associated Factors of Hypertension

In multilevel logistic analyses of risk factors of hypertension, older age, and comorbidities (central obesity, diabetes, dyslipidemia, or hyperuricemia) were associated with an increased risk of hypertension in both ethnic groups. In the Bouyei, non/ex-drinkers or light drinkers experienced significantly reduced risk than the harmful drinkers (OR 0.61, 95% CI 0.46–0.81 for non/ex-drinkers; OR 0.58, 95% CI 0.42–0.80 for light drinkers). In the Han, better education and having no family history of hypertension were observed to be associated with a decreased risk of hypertension (Table 3).

### 3.4. Associated Factors of Effective Hypertension Control in Treated Patients

When analyzing factors associated with effective control of hypertension, we restricted our analysis to patients under treatment. In the Bouyei, moderate physical activity emerged as a significant factor for effective control. Taken low physical activity as the reference, moderate physical activity was positively associated with improved control (OR 6.98, 95% CI 1.34–36.36) while high activity was not. In the Han, elderly people were more likely to have their BP adequately controlled than the young (OR 3.68, 95% CI 1.25–10.8) (Table 4).

## 4. Discussion

This study presented a picture of highly prevalent but poorly managed hypertension in Bouyei and Han ethnicities in Guizhou province. Awareness, treatment, and control were the three aspects of hypertension management in this study. Totally, 35.3% of Bouyei and 33.7% of Han were hypertensive. The age-sex standardized prevalence of hypertension in Bouyei was similar to that in Han. Among hypertensive Bouyei, 30.1% were aware of their hypertensive status with 19.7% received treatment, but only 3.6% achieved BP goal. Han had relatively better management of hypertension, with 40.2% awareness, 31.1% receiving treatment, and 9.9% under control. The age-sex standardized rates of awareness, treatment, and control were consistently lower in Bouyei than in Han. When comparing subgroups in age strata, Bouyei were found to have disproportionately lower rates of awareness, treatment, and control than Han in the older groups. However, such ethnic disparities disappeared in the younger groups, which might indicate an interaction between age and ethnicity. When analyzing hypertension risk in Bouyei and Han, some socio-demographic factors, lifestyle characteristics, and comorbidities were evident in associations with the risk of hypertension. In both ethnic groups, those of older age were more likely to develop hypertension compared to the young. Besides traditional risk factors like central obesity and metabolic disorders (diabetes and dyslipidemia), hyperuricemia emerged as another risk factor for hypertension. In the Bouyei, alcohol abstinence or light alcohol intake played beneficial roles in risk reduction. In the Han, higher education was in an inverse and graded association with the hypertension risk. Having no family history of hypertension was associated with reduced risk of hypertension. With respect to factors associated with effective control in treated patients, only age remained significant. Elderly Han were more likely to have their BP under control than young Han. In the Bouyei, moderate physical activity played a beneficial role in effective hypertension control while high activity did not.

Due to limited data on hypertension in Bouyei population and different population structures used for standardization, it is difficult to compare our results with those from the previous studies [10,18]. Even so, BP levels in our study are concordant with some studies. In the 2011 Guizhou provincial hypertension survey [19], the average SBP was 125.8 mmHg, a bit lower than 131.6 mmHg of pooled sample in the present study, and DBP (78.4 mmHg) was very close to our findings (78.3 mmHg). In agreement with many other hypertension surveys in middle or low-income regions in China and other countries [5,6,20,21,22], the present data displayed a dismal situation of high prevalence but low awareness, treatment, and control in the developing regions. Although the overall control rate of hypertension was strikingly low, elderly Han were found to have a significantly higher rate of control than the elderly Bouyei. Such ethnic discrepancy may relate to the healthcare inequalities and different economic status. In our data, Han people had higher income levels and were more economically advantaged. They lived mainly in urban regions and had better access to treatment than Bouyei who were mostly living in the mountainous regions. Regarding the aforementioned highly prevalent but worse managed hypertension in elderly Bouyei, management on hypertension in this fraction of population should be a major concern and tailored strategies need to be targeted toward this population.

In multivariate analyses on risk factors related to hypertension in Bouyei and Han, ageing population or people with central obesity, diabetes, dyslipidemia, or hyperuricemia were at higher risk of being hypertensive. Ageing is a well-established but unalterable risk factor for hypertension. Some modifiable factors were identified in each ethnic group, such as education and alcohol consumption, which could help to direct favorable lifestyle changes. Additionally, hyperuricemia emerged as a risk factor for hypertension, which was consistent with the findings in a Chinese prospective study [23] and a Japanese screened cohort [24]. Disordered uric acid was supposed to influence changes in renin and NOS1 in the pathogenesis of hypertension [25], and it was mentioned as a modifying and causal factor for human primary hypertension [26]. However, no significant association was observed between health insurance and hypertension in both ethnic groups. Our data showed that 98.8% of the Bouyei and 97.6% of the Han had their health insurance covered. The high proportions of health insurance in both hypertensive and normotensive participants could result in statistically insignificant differences. Such wide coverage was probably due to the expanding coverage of health insurance in the Reformed Program on Chinese health Care System [27].

When analyzing the outcomes of effective control in treated Bouyei and Han patients, the major associations with the outcome were physical activity and age. In Bouyei patients, the moderate intensity of physical activity produced beneficial effects on hypertension control. This finding was concordant with the results from an exercise intervention study conducted in hypertensive patients [28]. In the exercise trial, clinically significant reduction in BP was achieved through moderate intensity activity but not on graded reduction in BP when increasing the levels of exercise. The authors concluded that the amount of exercise required for BP reduction might be relatively small and attainable. In another study on exercise training in hypertensive rats, lower intensity of exercise training (55% maximal oxygen uptake) showed better effect on BP reduction than higher intensity (85% maximal oxygen uptake) [29]. Results from all the above studies demonstrated that an appropriate intensity of physical activity was crucial for the reduction of high BP. The mechanisms of a fall in BP through physical activity may be attributed to the immediate dilation of arteries after exercising and subsequently reducing the vascular resistance [30] or the decreased plasma renin activity and catecholamines combined with increased urinary sodium excretion [31]. The positive effect of moderate physical activity on BP reduction prompted a non-pharmacologic way in managing the hypertensive patients. To benefit general population, moderate physical exercise should be integrated into health education program for urban/rural households and communities. Health education is cost-effective in the prevention and control of hypertension [32] and can effectively increase public awareness of moderate exercise. In Han patients, older age was positively associated with effective hypertension control. This finding was in agreement with the results of other surveys conducted in China [33,34].

Our study is by far the largest health survey in Bouye ethnic minority. Standardized protocols and validated methods were applied simultaneously to Bouyei and Han, so to have provided comparable ethnic disparities in hypertension. Since a multistage stratified method was adopted to select participants from non-institutionalized population in communities, the sample was supposed to be representative in nature. The disparities in hypertension between Bouyei and Han could reflect the gap in health between minority groups and Han majority in southern China. Based on the sampling design and geographic concentration of ethnic groups, multilevel models with random intercepts were used for data interpretation and clustered effect was taken into consideration. The primary limitation of this study was that the temporal relationships between exposures and outcomes were unclear (e.g., the relationships between metabolic disorders and hypertension) due to the inherent weakness of cross-sectional studies. In the present study, no dietary information was obtained. Since it is difficult to accurately quantify the dietary assessment in large-scale population, sodium and other dietary intake that might be linked to hypertension were not collected in this Health Survey. Finally, individuals with chronic kidney disease (CKD) were unable to be diagnosed in this epidemiological survey. This may lead to an overestimation of control rate of hypertension in elderly patients since the BP goal for patients aged ≥60 years with CKD is <140/90 mmHg rather than 150/90 mmHg [16].

## 5. Conclusions

We hereby presented noteworthy findings about ethnic disparities in hypertension in two ways. First, prevalence rates were similarly high both in Bouyei and Han, but the rates of awareness, treatment, and control of hypertension were all substantially lower in Bouyei, particularly in the elderly population. This undesirable result reflects the need for planning and reasonable allocation of constrained health resources in the developing regions of South China and elderly Bouyei should be the target population. Secondly, favorable lifestyle modifications including avoidance of excessive alcohol consumption and high education could reduce the risk of hypertension. Moderate physical activity could be an effective non-pharmacologic intervention for Bouyei patients to control their BP. Tailored strategy needs to be targeted toward ethnic minorities.

## Figures and Tables

**Table 1:** Demographic and Health Characteristics of Bouyei and Han.

Variables	Bouyei (*n* = 2746)	Han (*n* = 2824)	*p*
No. (%) ^a^	–	–	–
Rural	2430 (88.5)	996 (35.3)	<0.0001
Men	1157 (42.1)	1140 (40.4)	0.1808
Education	–	–	–
Low	1655 (60.6)	930 (33.1)	<0.0001
Medium	797 (29.2)	1119(39.8)	
High	279 (10.2)	761 (27.1)	
Smoking, never	1841 (67.1)	1899 (67.6)	0.6979
Alcohol Consumption	–	–	–
Never/Ex-drinker	1642 (59.8)	1832 (65.2)	<0.0001
Light use	432 (15.7)	629 (22.4)	
Harmful use	672 (24.5)	350 (12.5)	
Recreational Activity	–	–	–
High	132 (4.9)	602 (21.5)	<0.0001
Moderate	142 (5.2)	418 (14.9)	
Low	2444 (89.9)	1780 (63.6)	
Occupational activity	–	–	–
High	1597 (58.3)	540 (19.2)	<0.0001
Moderate	161 (5.9)	282 (10.0)	
Low	982 (35.8)	1997 (70.8)	
Income	–	–	–
Low	1021 (37.5)	401 (14.4)	<0.0001
Low-middle	825 (30.3)	528 (18.9)	
Upper-middle	463 (17.0)	964 (34.5)	
High	414 (15.2)	898 (32.2)	
Health insurance, insured	2709 (98.8)	2752 (97.6)	0.0004
Family history, yes	316 (11.5)	878 (31.1)	<0.0001
Central obesity ^b^	408 (19.4)	939 (39.1)	<0.0001
Hyperuricemia ^c^	273 (10.0)	410 (14.7)	<0.0001
Median (IQR)	–	–	–
Age, year	50 (40,62)	48 (37,60)	<0.0001
Systolic BP, mmHg	129 (117,145)	127 (115,142)	<0.0001
Diastolic BP, mmHg	77 (70,86)	77 (70,86)	0.9695
Fasting glucose, mmol/L	4.9 (4.5,5.3)	4.9 (4.6,5.3)	0.0002
TC, mmol/L	4.8 (4.2,5.5)	4.9 (4.3,5.6)	0.0033
TG, mmol/L	1.1 (0.8,1.6)	1.3 (0.9,2.0)	<0.0001
LDL, mmol/L	2.6 (2.1,3.2)	2.8 (2.3,3.3)	<0.0001
HDL, mmol/L	1.6 (1.3,1.8)	1.4 (1.2,1.6)	<0.0001

^a^ No. indicates number; ^b^ Central obesity was averaged waist circumference ≥80 cm for women and ≥85 cm for men; ^c^ Hyperuricemia was set as uric acid in serum ≥416 umol/L for men and ≥357 umol/L for women.

**Table 2:** Prevalence and control of hypertension by ethnicity, sex, and age.

Age ^a^ and Sex	Prevalence, No. (%)		Awareness among Hypertension, No. (%)		Treatment among Hypertension, No. (%)		Control among Hypertension, No. (%)	
	Bouyei	Han	Bouyei	Han	Bouyei	Han	Bouyei	Han
Age	–	–	–	–	–	–	–	–
<50 year	237 (17.9)	261 (16.8)	54 (22.8)	69 (26.4)	35 (14.8)	43 (16.5)	5 (2.1)	6 (2.3)
≥50 year	731 (51.5)	691 (54.6)	237 (32.4)	314 (45.4)	156 (21.3)	253 (36.6)	30 (4.1)	88 (12.7)
Sex	–	–	–	–	–	–	–	–
Men	479 (41.4)	427 (37.5)	145 (30.3)	171 (40.1)	89 (18.6)	124 (29.0)	15 (3.1)	41 (9.6)
Women	489 (30.8)	525 (31.2)	146 (29.9)	212 (40.4)	102 (20.9)	172 (32.8)	20 (4.1)	53 (10.1)
All	968 (35.3)	952 (33.7)	291 (30.1)	383 (40.2)	191 (19.7)	296 (31.1)	35 (3.6)	94 (9.9)

^a^ Age was dichotomized into <50 and ≥50 years since hypertension prevalence grew rapidly in 50 s (see Figure S2, Table S1, which portrayed the changes of hypertension prevalence over age span in sex-ethnic strata and crude rates of prevalence, awareness, treatment, and control in age-sex-ethnic strata).

**Table 3:** Results of Multilevel Logistic Models for Hypertension in Bouyei and Han.

Variables	Bouyei		Han	
	OR (95% CI)	*p*	OR (95% CI)	*p*
Location (Ref = rural)	0.99 (0.63, 1.56)	0.9647	1.08 (0.75, 1.56)	0.6750
Age (Ref ≤ 50 years)	5.23 (4.11, 6.65)	<0.0001	4.93 (3.91, 6.22)	<0.0001
Education (Ref = low)	–	–	–	–
High	0.79 (0.50, 1.24)	0.2985	0.64 (0.46, 0.90)	0.0096
Medium	0.85 (0.66, 1.11)	0.2292	0.74 (0.57, 0.96)	0.0229
Insurance (Ref = no)	0.75 (0.26, 2.15)	0.5963	2.18 (0.89, 5.38)	0.0900
Family history (Ref = yes)	0.89 (0.64, 1.24)	0.4795	0.57 (0.46, 0.71)	<0.0001
Physical activity (Ref = low)	–	–	–	–
High	1.01 (0.79, 1.29)	0.9514	1.17 (0.92, 1.48)	0.1953
Moderate	0.84 (0.56, 1.26)	0.3899	1.00 (0.75, 1.33)	0.9723
Alcohol drinker (Ref = harmful)	–	–	–	–
Non/ex drinker	0.55 (0.42, 0.72)	<.0001	0.75 (0.54, 1.03)	0.0805
Light drinker	0.56 (0.41, 0.77)	0.0004	0.77 (0.54, 1.08)	0.1315
Smoking (Ref = ever)	1.00 (0.78, 1.28)	0.9886	0.89 (0.70, 1.12)	0.3171
Central obesity (Ref = no)	1.96 (1.49, 2.58)	<0.0001	2.31 (1.87, 2.86)	<0.0001
Diabetes (Ref = no) ^a^	1.81 (1.00, 3.27)	0.0489	2.08 (1.42, 3.04)	0.0002
Dyslipidemia (Ref = no) ^b^	1.71 (1.33, 2.21)	<0.0001	1.32 (1.07, 1.65)	0.0117
Hyperuricemia (Ref = no)	2.19 (1.57, 3.05)	<0.0001	1.98 (1.49, 2.63)	<0.0001

Ref indicates reference. All the variables were included as fixed effects in multilevel models; ^a^ Diabetes was defined as fasting glucose ≥7.0 mmol/L or reported diagnosis; ^b^ Dyslipidemia was defined as TC ≥ 6.22 mmol/L, LDL ≥ 4.14 mmol/L, TG ≥ 2.26 mmol/L, or HDL < 1.04 mmol/L.

**Table 4:** Results of Multilevel Logistic Models for Hypertension Control in Treated Bouyei and Han.

Variables	Bouyei ^a^		Han	
	OR (95% CI)	*p*	OR (95% CI)	*p*
Age (Ref ≤ 50 years)	0.93 (0.26, 3.37)	0.9081	3.68 (1.25, 10.80)	0.0182
Sex (Ref = women)	0.87 (0.20, 3.87)	0.8560	1.95 (0.75, 5.03)	0.1681
Family history (Ref = yes)	1.75 (0.43, 7.13)	0.4384	0.63 (0.35, 1.14)	0.1263
Physical activity (Ref = low)	–	–	–	–
High	1.87 (0.71, 4.93)	0.4998	1.85 (0.91, 3.73)	0.0880
Moderate	6.98 (1.34, 36.36)	0.0371	2.07 (0.82, 5.20)	0.1215
Alcohol drinker (Ref = harmful)	–	–	–	–
Non/ex drinker	1.88 (0.53, 6.70)	0.2564	1.92 (0.68, 5.38)	0.2164
Light drinker	1.01 (0.20, 5.20)	0.6688	1.31 (0.39, 4.37)	0.6580
Smoking (Ref = ever)	0.51 (0.11, 2.28)	0.3780	1.80 (0.69, 4.70)	0.2281
Central obesity (Ref = no)	1.25 (0.47, 3.36)	0.6554	1.03 (0.55, 1.92)	0.9244

Ref indicates reference. All the variables were included as fixed effects in multilevel models. ^a^ Multivariate logistic regression was used, as there was no random effect in Bouyei patients under treatment.

## Supplementary Materials

The following are available online at www.mdpi.com/1660-4601/13/2/233/s1, Figure S1: Flow chart of Data Management, Figure S2: Crude prevalence of hypertension by Ethnicity, Sex, and Age, Table S1: Crude rates of prevalence, awareness, treatment, and control by Ethnicity, Sex, and Age, Table S2: Age-, sex-adjusted rates of prevalence, awareness, treatment, and control between Ethnic groups.

## Abbreviations

The following abbreviations are used in this manuscript:

HBP: High Blood Pressure
BP: Blood Pressure
SBP: Systolic Blood Pressure
DBP: Diastolic Blood Pressure
IQR: Interquartile range
TC: Total Cholesterol
TG: Triglyceride
LDL: Low-Density Lipoproteins Cholesterol
HDL: High-Density Lipoproteins Cholesterol
CKD: Chronic Kidney Disease
