# Correction: Dong, F.; et al. Disparities in Hypertension Prevalence, Awareness, Treatment and Control between Bouyei and Han: Results from a Bi-Ethnic Health Survey in Developing Regions from South China. *Int. J. Environ. Res. Public Health* 2016, *13*, 233

**DOI:** 10.3390/ijerph13090922

**Published:** 2016-09-17

**Authors:** Fen Dong, Dingming Wang, Li Pan, Yangwen Yu, Ke Wang, Ling Li, Li Wang, Tao Liu, Xianjia Zeng, Liangxian Sun, Guangjin Zhu, Kui Feng, Biao Zhang, Ke Xu, Xinglong Pang, Ting Chen, Hui Pan, Jin Ma, Yong Zhong, Bo Ping, Guangliang Shan

**Affiliations:** 1 Department of Epidemiology and Biostatistics, Institute of Basic Medical Sciences Chinese Academy of Medical Sciences, School of Basic Medicine Peking Union Medical College, Beijing 100005, China; fionarab@163.com (F.D.); panli1716@163.com (L.P.); wangkehope@126.com (K.W.); wangli0528@vip.sina.com (L.W.); zxj28@sohu.com (X.Z.); zhuguangjinpumc@126.com (G.Z.); fengkui@sina.com (K.F.); sljzhangbiao11@126.com (B.Z.); 2 Guizhou Center for Disease Control and Prevention, Guizhou 550004, China; wangdingm123@sina.com (D.W.); yuyangweny@163.com (Y.Y.); gpicdp@163.com (L.L.); liutao9099@163.com (T.L.); slx1087@163.com (L.S.); 3 Department of Endocrinology, Peking Union Medical College Hospital, Chinese Academy of Medical Sciences & Peking Union Medical College, Beijing 100730, China; zjwzxuke@126.com (K.X.); pxl19870610@163.com (X.P.); panhui20111111@163.com (H.P.); 4 Department of Ophthalmology, Peking Union Medical College Hospital, Chinese Academy of Medical Sciences & Peking Union Medical College, Beijing 100730, China; ct19870629@hotmail.com (T.C.); majin1912@163.com (J.M.); yzhong_eye@163.com (Y.Z.); 5 Longli Center for Disease Control and Prevention, Guizhou 551200, China; gzlljkpb@126.com

The authors wish to add the following amendments and corrections to their paper published in the International Journal of Environmental Research and Public Health [1]. 

Page 7, Section 3.3. Associated factors of hypertension: the odds ratio (OR) and 95% confidence interval (CI) for non/ex-drinkers and light drinkers are not consistent with the correspondent numbers in Table 3. The correct OR and 95% CI should be:

## 3.3. Associated Factors of Hypertension

In multilevel logistic analyses of risk factors of hypertension, older age and comorbidities (central obesity, diabetes, dyslipidemia, or hyperuricemia) were associated with an increased risk of hypertension in both ethnic groups. In the Bouyei, non/ex-drinkers or light drinkers experienced significantly reduced risk than the harmful drinkers (OR 0.55, 95% CI 0.42–0.72 for non/ex-drinkers; and OR 0.56, 95% CI 0.41–0.77 for light drinkers). In the Han, better education and having no family history of hypertension were observed to be associated with a decreased risk of hypertension (Table 3).

**Table 3:** Results of Multilevel Logistic Models for Hypertension in Bouyei and Han.

Variables	Bouyei		Han	
	OR (95% CI)	*p*	OR (95% CI)	*p*
Location (Ref = rural)	0.99 (0.63, 1.56)	0.9647	1.08 (0.75, 1.56)	0.6750
Age (Ref ≤ 50 years)	5.23 (4.11, 6.65)	<0.0001	4.93 (3.91, 6.22)	<0.0001
Education (Ref = low)	–	–	–	–
High	0.79 (0.50, 1.24)	0.2985	0.64 (0.46, 0.90)	0.0096
Medium	0.85 (0.66, 1.11)	0.2292	0.74 (0.57, 0.96)	0.0229
Insurance (Ref = no)	0.75 (0.26, 2.15)	0.5963	2.18 (0.89, 5.38)	0.0900
Family history (Ref = yes)	0.89 (0.64, 1.24)	0.4795	0.57 (0.46, 0.71)	<0.0001
Physical activity (Ref = low)	–	–	–	–
High	1.01 (0.79, 1.29)	0.9514	1.17 (0.92, 1.48)	0.1953
Moderate	0.84 (0.56, 1.26)	0.3899	1.00 (0.75, 1.33)	0.9723
Alcohol drinker (Ref = harmful)	–	–	–	–
Non/ex drinker	0.55 (0.42, 0.72)	<0.0001	0.75 (0.54, 1.03)	0.0805
Light drinker	0.56 (0.41, 0.77)	0.0004	0.77 (0.54, 1.08)	0.1315
Smoking (Ref = ever)	1.00 (0.78, 1.28)	0.9886	0.89 (0.70, 1.12)	0.3171
Central obesity (Ref = no)	1.96 (1.49, 2.58)	<0.0001	2.31 (1.87, 2.86)	<0.0001
Diabetes (Ref = no) ^a^	1.81 (1.00, 3.27)	0.0489	2.08 (1.42, 3.04)	0.0002
Dyslipidemia (Ref = no) ^b^	1.71 (1.33, 2.21)	<0.0001	1.32 (1.07, 1.65)	0.0117
Hyperuricemia (Ref = no)	2.19 (1.57, 3.05)	<0.0001	1.98 (1.49, 2.63)	<0.0001

Ref indicates reference. All the variables were included as fixed effects in multilevel models; ^a^ Diabetes was defined as fasting glucose ≥ 7.0 mmol/L or reported diagnosis; ^b^ Dyslipidemia was defined as TC ≥ 6.22 mmol/L, LDL ≥ 4.14 mmol/L, TG ≥ 2.26 mmol/L, or HDL < 1.04 mmol/L.

The authors would like to apologize for any inconvenience caused to the readers by these changes.
