# Supplementary material for: Disparities in Hypertension Prevalence, Awareness, Treatment and Control between Bouyei and Han: Results from a Bi-Ethnic Health Survey in Developing Regions from South China
Source: Int J Environ Res Public Health. 2016 Feb 19;13(2):233. doi: 10.3390/ijerph13020233 (PMC4772253; doi:10.3390/ijerph13020233)
Supplement: Supplementary file 1 [file ijerph-13-00233-s001.pdf]

Supplementary Materials: Disparities in Hypertension Prevalence, Awareness, Treatment and Control between Bouyei and Han: Results from a Bi-Ethnic Health Survey in Developing Regions from South China

Fen Dong [1], Dingming Wang, Li Pan, Yangwen Yu, Ke Wang, Ling Li, Li Wang, Tao Liu, Xianjia Zeng, Liangxian Sun, Guangjin Zhu, Kui Feng, Biao Zhang, Ke Xu, Xinglong Pang, Ting Chen, Hui Pan, Jin Ma, Yong Zhong, Bo Ping and Guangliang Shan

Table S1. Crude rates of prevalence, awareness, treatment and control by Ethnicity, Sex, and Age [a].

Ethnicity, Sex and Age [a]	Percentage, N. (%)			
	Prevalence among Participants	Awareness among Hypertension	Treatment among Hypertension	Control among Hypertension
Bouyei Men	–	–	–	–
20 to 29	7 (8.05)	1 (14.29)	0 (0.00)	0 (0.00)
30 to 39	39 (20.74)	6 (15.38)	1 (2.56)	0 (0.00)
40 to 49	86 (29.55)	26 (30.23)	19 (22.09)	3 (3.49)
50 to 59	118 (49.17)	36 (30.51)	19 (16.1)	4 (3.39)
60 to 69	142 (61.47)	47 (33.1)	30 (21.13)	4 (2.82)
70 to 80	87 (72.50)	29 (33.33)	20 (22.99)	4 (4.60)
Bouyei Women	–	–	–	–
20 to 29	2 (2.44)	1 (50)	0 (0.00)	0 (0.00)
30 to 39	27 (9.38)	0 (0.00)	0 (0.00)	0 (0.00)
40 to 49	76 (19.49)	20 (26.32)	15 (19.74)	2 (2.63)
50 to 59	130 (37.46)	40 (30.77)	25 (19.23)	3 (2.31)
60 to 69	167 (50.15)	55 (32.93)	46 (27.54)	10 (5.99)
70 to 80	87 (58.39)	30 (34.48)	16 (18.39)	5 (5.75)
Han Men	–	–	–	–
20 to 29	11 (10.78)	0 (0.00)	0 (0.00)	0 (0.00)
30 to 39	34 (14.98)	7 (20.59)	2 (5.88)	0 (0.00)
40 to 49	87 (30.96)	28 (32.18)	19 (21.84)	2 (2.30)
50 to 59	110 (47.83)	47 (42.73)	32 (29.09)	11 (10.00)
60 to 69	121 (58.17)	55 (45.45)	45 (37.19)	14 (11.57)
70 to 80	64 (69.57)	34 (53.13)	26 (40.63)	14 (21.88)
Han Women	–	–	–	–
20 to 29	5 (2.40)	0 (0.00)	0 (0.00)	0 (0.00)
30 to 39	30 (9.49)	7 (23.33)	5 (16.67)	2 (6.67)
40 to 49	94 (22.17)	27 (28.72)	17 (18.09)	2 (2.13)
50 to 59	144 (43.77)	60 (41.67)	47 (32.64)	12 (8.33)
60 to 69	169 (60.14)	86 (50.89)	74 (43.79)	25 (14.79)
70 to 80	83 (65.87)	32 (38.55)	29 (34.94)	12 (14.46)

[a] Age was grouped by 10 years.

Table S2. Age-, sex-adjusted rates of prevalence, awareness, treatment and control between Ethnic groups.

Age and Sex	%(95% CI)											
	**Prevalence**			**Awareness [a]**			**Treatment [a]**			**Control Rate [a]**		
	**Bouyei**	**Han**	***p***	**Bouyei**	**Han**	***p***	**Bouyei**	**Han**	***p***	**Bouyei**	**Han**	***p***
Age	–	–	–	–	–	–	–	–	–	–	–	–
<50 years	16.3 (13.37,19.72)	16.27 (13.35,19.69)	0.9861	22.75 (17.56,28.93)	26.12 (20.77,32.28)	0.3919	14.86 (10.8,20.1)	16.45 (12.36,21.56)	0.6287	3.07 (1.05,8.64)	2.23 (0.73,6.60)	0.6417
≥50 years	51.68 (45.68,57.63)	52.11 (45.77,58.39)	0.8726	32.87 (28.04,38.10)	42.77 (36.99,48.76)	0.0017	22.68 (18.21,27.88)	32.72 (26.81,39.24)	0.0009	4.75 (2.93,7.63)	11.13 (7.38,16.45)	0.0009
Sex	–	–	–	–	–		–	–	–	–	–	–
Men	36.29 (30.26,42.79)	33.78 (27.80,40.32)	0.3291	26.84 (21.95,32.36)	35.5 (29.63,41.85)	0.0096	17.17 (13.07,22.24)	25.23 (19.78,31.58)	0.0070	2.35 (1.13,4.82)	5.95 (3.22,10.73)	0.0071
Women	25.6 (22.14,29.40)	28.52 (24.67,32.71)	0.1415	26.1 (20.93,32.04)	33.65 (27.58,40.31)	0.0230	18.05 (13.59,23.58)	24.82 (19.18,31.46)	0.0229	3.03 (1.54,5.87)	6.26 (3.47,11.04)	0.0253

[a] Awareness, treatment and control rates among hypertensive participants.

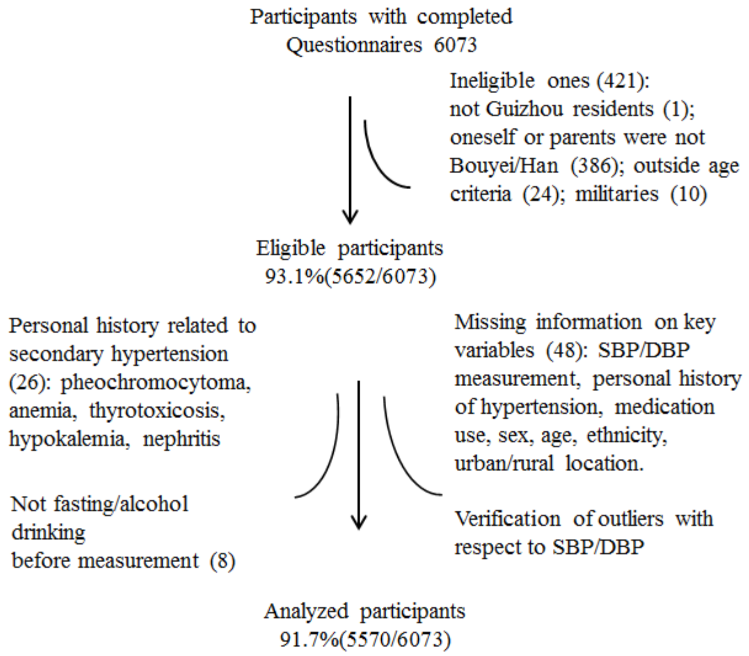

Figure S1. Flow chart of Data Management.

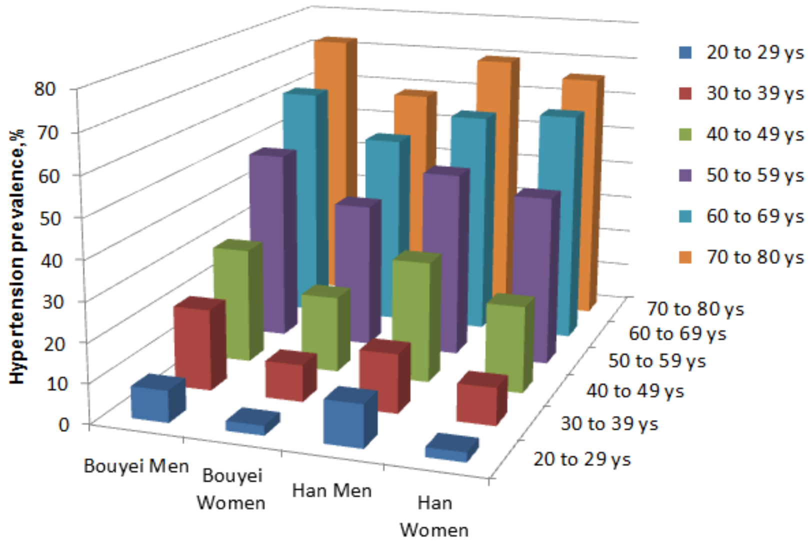

Figure S2. Crude prevalence of hypertension by Ethnicity, Sex, and Age.

© 2016 by the authors; licensee MDPI, Basel, Switzerland. This article is an open access article distributed under the terms and conditions of the Creative Commons by Attribution (CC-BY) license (http://creativecommons.org/licenses/by/4.0/).
